# Recognizing animal personhood in compassionate conservation

**DOI:** 10.1111/cobi.13494

**Published:** 2020-05-18

**Authors:** Arian D. Wallach, Chelsea Batavia, Marc Bekoff, Shelley Alexander, Liv Baker, Dror Ben‐Ami, Louise Boronyak, Adam P. A. Cardilin, Yohay Carmel, Danielle Celermajer, Simon Coghlan, Yara Dahdal, Jonatan J. Gomez, Gisela Kaplan, Oded Keynan, Anton Khalilieh, Helen Kopnina, William S. Lynn, Yamini Narayanan, Sophie Riley, Francisco J. Santiago‐Ávila, Esty Yanco, Miriam A. Zemanova, Daniel Ramp

**Affiliations:** ^1^ Centre for Compassionate Conservation, Faculty of Science University of Technology Sydney Sydney NSW 2007 Australia; ^2^ Department of Forest Ecosystems and Society Oregon State University Corvallis OR 97331 U.S.A.; ^3^ Ecology and Evolutionary Biology University of Colorado Boulder CO 80309 U.S.A.; ^4^ Department of Geography University of Calgary Calgary AB T2N 1N4 Canada; ^5^ Animal Behavior and Conservation Program Hunter College CUNY New York NY U.S.A.; ^6^ Compassionate Conservation Middle East, Steinhardt Museum of Natural History Tel Aviv University Tel Aviv Israel; ^7^ Institute for Sustainable Futures University of Technology Sydney Sydney NSW 2007 Australia; ^8^ Faculty of Science Engineering and Built Environment Deakin University Waurn Ponds VIC 3216 Australia; ^9^ Division of Environmental, Water and Agricultural Engineering Technion Israel Institute of Technology Haifa 32000 Israel; ^10^ Department of Sociology and Social Policy, Faculty of Arts and Social Sciences The University of Sydney Sydney NSW 2006 Australia; ^11^ School of Computing and Information Systems, Melbourne School of Engineering The University of Melbourne Parkville VIC 3010 Australia; ^12^ Nature Palestine West Bank Palestine; ^13^ Departamento de Ciencias Básicas Universidad Nacional de Luján Rutas 5 y 7 Luján 6700 Argentina; ^14^ School of Science & Technology University of New England Armidale NSW 2351 Australia; ^15^ Dead Sea & Arava Science Centre Central Arava Branch Hatzeva Israel; ^16^ The Hague University of Applied Sciences, International Business Johanna Westerdijkplein 75 EN Den Haag 2521 the Netherlands; ^17^ George Perkins Marsh Institute Clark University Worcester MA 01710 U.S.A.; ^18^ School of Humanities and Social Sciences, Faculty of Arts and Education Deakin University Melbourne VIC 3125 Australia; ^19^ Faculty of Law University of Technology Sydney Sydney NSW 2007 Australia; ^20^ Carnivore Coexistence Lab, Nelson Institute for Environmental Studies University of Wisconsin‐Madison Madison WI 53706 U.S.A.

**Keywords:** animal ethics, biodiversity, conservation ethics, human exceptionalism, nativism, biodiversidad, ética animal, ética de la conservación, excepcionalidad humana, nativismo, 动物伦理, 生物多样性, 保护伦理, 人类例外论, 本土主义

## Abstract

Compassionate conservation is based on the ethical position that actions taken to protect biodiversity should be guided by compassion for all sentient beings. Critics argue that there are 3 core reasons harming animals is acceptable in conservation programs: the primary purpose of conservation is biodiversity protection; conservation is already compassionate to animals; and conservation should prioritize compassion to humans. We used argument analysis to clarify the values and logics underlying the debate around compassionate conservation. We found that objections to compassionate conservation are expressions of human exceptionalism, the view that humans are of a categorically separate and higher moral status than all other species. In contrast, compassionate conservationists believe that conservation should expand its moral community by recognizing all sentient beings as persons. Personhood, in an ethical sense, implies the individual is owed respect and should not be treated merely as a means to other ends. On scientific and ethical grounds, there are good reasons to extend personhood to sentient animals, particularly in conservation. The moral exclusion or subordination of members of other species legitimates the ongoing manipulation and exploitation of the living worlds, the very reason conservation was needed in the first place. Embracing compassion can help dismantle human exceptionalism, recognize nonhuman personhood, and navigate a more expansive moral space.

## Abstract 2

*Article Impact Statement*: The debate about compassionate conservation is about whether to recognize nonhuman personhood.

## Abstract (es)

Reconocimiento de la Calidad de Persona en los Animales dentro de la Conservación Compasiva

**Resumen**

La conservación compasiva está basada en la posición ética que parte de que las acciones tomadas para proteger a la biodiversidad deberían estar dirigidas por la compasión por todos los seres sintientes. Los críticos de esta postura argumentan que hay tres razones nucleares por las que el daño a los animales es aceptable dentro de los programas de conservación: el principal motivo de la conservación es la protección de la biodiversidad; la conservación ya es compasiva con los animales; y la conservación debería priorizar la compasión hacia los humanos. Usamos un análisis de argumentos para aclarar los valores y la lógica subyacentes al debate en torno a la conservación compasiva. Encontramos que el rechazo a la conservación compasiva es una expresión de la excepcionalidad humana, la visión de que los humanos están en un nivel categóricamente separado y de mayor moral que todas las demás especies. Por el contrario, los conservacionistas compasivos creen que la conservación debería expandir su comunidad moral al reconocer a todos los seres sintientes como personas. La calidad de persona, en un sentido ético, implica que el individuo merece respeto y no debería ser tratado solamente como un medio para otros fines. Si hablamos desde fundamentos científicos y éticos, existen muy buenas razones para extender la calidad de persona a todos los animales sintientes, particularmente en la conservación. La exclusión moral o la subordinación de los miembros de otras especies justifica la continua manipulación y explotación de los seres vivos, la justa razón por la que necesitamos de la conservación desde el principio. La aceptación de la compasión nos puede ayudar a desmantelar la excepcionalidad humana, a reconocer la calidad de persona no humana y a navegar un espacio moral más expansivo.

## 摘要

同情心保护基于这样一种伦理立场, 即生物多样性保护行动的指导原则应为对众生的同情心。而批评者认为在保护项目中可以接受对动物的伤害, 其三个核心原因是:保护的主要目的是保护生物多样性;保护动物已经是同情心的体现;保护应该优先考虑对人类的同情心。本研究使用了论点分析来阐明关于同情心保护的辩论背后的价值和逻辑。我们发现, 对同情心保护的反对是人类例外论的表现, 这种观点认为人类独立于其它所有物种且拥有更高的道德地位。相比之下, 同情心保护主义者则认为, 在保护中应承认众生与人类平等的存在, 以扩大其道德共同体的范围。在伦理意义上, 人格性意味着个体应该被尊重, 而不应该仅仅被当作达到其它目的的手段。而从科学和伦理的角度来看, 我们有充分的理由将人格性扩展到有感知能力的动物身上, 特别是在保护当中。对其它物种的道德排斥或从属化导致了人们长期以来对生命世界操纵和剥削的合法化, 而这也正是目前需要进行保护的首要原因。积极拥抱同情心可以帮助消除人类例外论, 认识到非人类生命的人格性, 并导向更广阔的道德空间。 **【翻译: 胡怡思; 审校: 聂永刚】**

## Introduction

Western culture traditionally regarded humans as exceptional among all animals (Rose 2011). This belief was challenged by scientific and philosophical breakthroughs confirming Charles Darwin's view that “there is no fundamental difference” between humans and other animals (Darwin 1871). Today, it is beyond dispute that many animals are sentient beings (Low et al. 2012; Bekoff & Pierce 2017). Much has changed since the 1960s, when primatologist Jane Goodall was castigated for ascribing chimpanzees with personalities and feelings (Goodall 1998). Despite this, even those nonhuman animals with recognized mental, emotional, and social sophistication (such as mammals, birds, and cephalopods) are still readily treated as means to human ends (Midgley 1985). Conservation is no exception (Wallach et al. 2018).

Although conservation was founded on a uniquely expansive ethic that recognizes the intrinsic value of the living world (Batavia & Nelson 2017), nonhuman animals do not necessarily meet a better fate in the hands of conservationists than in any other hands (Ramp & Bekoff 2015; Wallach et al. 2018). Conservationists often embody deep concern, even love, for wildlife and nature. Yet, this can be a “violent love” (Srinivasan & Kasturirangan 2017). Systemic harm of sentient animals in conservation is enabled by 3 ethical orientations: collectivism (or holism)—the belief that species matter more than individuals; instrumentalism—the treatment of an entity as a means to an end; and nativism—the view that populations established by humans are unnatural (Wallach et al. 2018). These orientations drive conservation practices that use “authoritarian management and control measures” (Bhattacharyya & Larson 2014) and that regard nonhuman animals as “instances of their type” (Vucetich & Nelson 2007) and as “killable” (Haraway 2013) “objects” (Nussbaum 1995).

Compassionate conservation, in contrast, recognizes that the interests and agency of all sentient beings should be protected in conservation practice (Ramp & Bekoff 2015; Wallach et al. 2018). In other words, sentient beings are persons. Personhood, in an ethical sense, implies an entity is owed respect and should not be treated as a means to other ends (Midgley 1985; Dayan 2018). Many traditions (including hunting‐based cultures) have long understood the world as animated with multitudes of persons with whom humans form kinship relations (e.g., Rose 2011; Hill 2013; Robinson 2014). Western tradition, however, has largely restricted the notion of personhood to humans, an expression of human exceptionalism that maintains humans as a categorically separate and inherently superior class of being (Plumwood 1993). It is not our purpose to dispute this view, per se, but to offer an alternative; namely, all sentient beings are persons when viewed through the lens of compassion.

The proposition that compassion for sentient beings should inform and where necessary redirect conservation goals and practices has generated intense debate. Recently, a series of critiques of compassionate conservation unfolded in *Conservation Biology* and included a Conservation Focus “Debating Compassion in Conservation Science” (2019, Volume 33, Issue 4). We considered the main statements made in opposition to compassionate conservation in 5 essays that responded to Wallach et al. (2015) and Wallach et al. (2018): Russell et al. (2016), Driscoll and Watson (2019), Hampton et al. (2019), Hayward et al. (2019), and Oommen et al. (2019) (hereafter critiques or critics). We condensed the critiques into 3 core reasons to reject compassionate conservation and support lethal and invasive conservation practices: the primary purpose (raison d'être) of conservation is biodiversity protection; conservation is already compassionate to animals; and conservation should prioritize compassion to humans (Table 1). We developed these reasons into formal arguments, which allowed us to clarify the values and logic underlying the debate around compassionate conservation. Formal arguments are composed of a set of premises (P) leading to a conclusion (C). An argument is sound when it meets 2 conditions: it is valid, meaning its conclusion necessarily follows from its premises, and its premises are true or appropriate, meaning the empirical premises are factually accurate and the ethical premises are consistently defensible (Hughes et al. 2010).

**Table 1:** Critic's reasons and example statements to reject compassionate conservation and maintain lethal and invasive conservation programs

Reasons	Example statements
The primary purpose of conservation is biodiversity protection.	Russell et al. (2016): “[Preventing] endangered species from going extinct… is the foundation of conservation biology.” “Restoration of… natural processes is at the core of the duty conservation biologists assume.” “[T]he goal [of lethal control of introduced species] is to reestablish natural ecological processes.” Driscoll and Watson (2019): “We, as conservation scientists, as ethical humans, want to preserve diversity. We want to preserve function. We want to preserve systems.” “[Arguments] ruling out culling invasive alien species… [is] squarely in the realm of science denialism.” Hayward et al. (2019): “[C]oncern for individual animals… [is appropriate] only to the extent that it is consistent with landscape‐level methods of protecting native biodiversity.” “Conservationists generally support [harming individual animals] because, at times, intervention is required.”
Conservation is already compassionate to animals.	Argument 1 Russell et al. (2016): “Where invasive predators are killed to achieve conservation goals, we believe this action can stem from compassion for all of the ecosystem, its species, the individuals being protected, and the invasive animals themselves.” “In some cases, lethal control is the most ethical and compassionate course of action.” Driscoll and Watson (2019): “Compassionate conservation is not compassionate.” Hampton et al. (2019): “[W]hen lethal control is performed [to best practice] animal welfare outcomes are in most cases superior to alternative management options.” Hayward et al. (2019): “Compassion (or, less specifically, concern for individual animal welfare) has already become an important aspect of best practices in conservation.” Argument 2 Hampton et al. (2019): “Under consequentialist approaches, contentious actions, such as killing, are considered ethically permissible if, when compared with alternative actions, they deliver a better balance of positive versus negative effects.” Driscoll and Watson (2019): “[Suffering associated with introduced species] is distinct from suffering and death of native species in natural ecosystems that are both an outcome of, and integral to, natural evolutionary processes.” “The suffering [compassionate conservationists] seek to prevent by adhering to virtue ethics leads to worse suffering and death.” Hayward et al. (2019): “[D]oing no harm to introduced [animals] results in more harm being done to more individual animals. Yet stopping the lethal control of invasive mammals, despite the inordinate amount of suffering they inflict on other animals, is a cardinal concern of compassionate conservationists.” “The methods used by professionals to kill animals for conservation purposes will almost always be more humane and compassionate than the methods used by animals to kill each other.”
Conservation should prioritize compassion to humans.	Hampton et al. (2019): “These positive effects [of killing] may be… [including] desirable outcome for humans through harvesting, improved quality of drinking water, [and] reduced vehicle collisions.” Oommen et al. (2019): “[Compassionate conservation] focuses on the well‐being of individual wild animals without adequately considering the well‐being or worldviews of [humans].” “The practical human costs of overplaying the moral salience of sentience and sapience in nonhuman animals are non‐trivial.” “[P]rograms that manage entire populations, species, or habitats based on consumptive sustainable use [should] be supported.” “[One should consider] the consequences of conservation action on human well‐being.” “Conservationists should not presume that one set of anthropomorphized, culturally specific values is universally applicable to all and independent of regional factors or local politics”

## Response to Claim that the Primary Purpose of Conservation is Biodiversity Protection

The global conservation community shares the beliefs that nature has intrinsic value and the role of conservation is to enable the flourishing of Earth's diversity of life (Sandbrook et al. 2019). However, precisely which and how living entities should be valued and protected remain complex and contested ethical questions. Compassionate conservationists recognize the intrinsic value of individuals alongside that of ecological collectives (also, ecological wholes) and call to avoid deliberately harming sentient beings in conservation programs (e.g., Ramp & Bekoff 2015; Wallach et al. 2018). Critics respond that there are situations in which lethal and invasive practices are necessary to protect biodiversity and prevent extinctions. For example, Russell et al. (2016) “believe lethal control of invasive predators is justified when it will reverse [their] negative impacts… on native species and ecosystems.” The first line of reasoning invoked to contest compassionate conservation can be summarized as follows (Table 1):

P1. Conservation actions that harm animals can be necessary to protect biodiversity.
C. Therefore, conservation actions that harm animals can be appropriate.

Critics tend to focus exclusively on the truthfulness of P1, assuming that if it can be verified, the conclusion necessarily follows (e.g., Driscoll & Watson 2019). This inference is invalid. Although descriptive conclusions (e.g., elephants are mammals) require only descriptive or empirical premises, prescriptive conclusions (e.g., elephants should be protected) require both descriptive and prescriptive premises. Thus, P1 by itself does not lead to C (Nelson et al. 2016). To be valid, the argument must include at least one ethical premise. For example:

P1. The goal of conservation is to protect biodiversity. (ethical)
P2. Actions that are necessary to achieve the goal of conservation are appropriate. (ethical)
P3. Conservation actions that harm animals can be necessary to protect biodiversity. (empirical)
C. Therefore, conservation actions that harm animals can be appropriate.

This argument is valid, but additional specifications are required to more accurately represent the critiques of compassionate conservation (Table 1). As expressed by critics, the goal of protecting biodiversity (P1) is usually limited to the protection of native species (Table 1), which are considered “of more value to their ecosystems than non‐native species” (Hayward et al. 2019). Additionally, in P2 and P3, critics do not intend to include humans among the animals available to be severely harmed to protect biodiversity (Table 1). To reflect these qualifications, the argument can be expanded:

P1. The goal of conservation is to protect biodiversity. (ethical)
P2. Protecting biodiversity means protecting native species. (ethical)
P3. Actions directed toward nonhuman animals that are necessary to achieve the goal of conservation are appropriate. (ethical)
P4. Conservation actions that harm nonhuman animals can be necessary to protect native species. (empirical)
C. Therefore, conservation actions that harm nonhuman animals can be appropriate.

This argument encapsulates the first key objection to compassionate conservation (Table 1). Premise 1 is both descriptive and ethical. It articulates what conservation aims to achieve (protect biodiversity) and, in so doing, identifies something considered of value (biodiversity). Premise 1 can be considered true and appropriate, and a starting point of agreement between critics and supporters of compassionate conservation. However, the ethical positions expressed in P2 and P3 are contestable.

Premise 2 reflects nativist and collectivist orientations by stipulating that only native species are worthy of conservation concern. Nativism is an influential but contested view (e.g., Chew & Hamilton 2011; Bhattacharyya & Larson 2014; Sandbrook et al. 2019; Gbedomon et al. 2020) that classifies and values nonhuman species based on their association, or lack thereof, with (usually Western) humans (Marris 2013). Collectivism, in turn, subsumes the value of individual nonhuman lives (both native and not) to the value of their species. Premise 3 then represents the instrumentalist assertion that measures directed against nonhuman animals are justified when necessary to achieve the assumed greater good of conservation (and when following accepted animal welfare protocols) (Table 1).

Premises 2 and 3, and their underlying orientations, highlight a core fissure between compassionate conservation and its critics. By advocating objectifying and lethal methods, critics of compassionate conservation withhold the ethical status of personhood from sentient nonhuman animals, even those with known cognitive and emotional sophistication. Compassionate conservationists, in contrast, take seriously that all sentient beings are persons, who should not be reduced to symbols of anthropogenic influence or treated as instances of a type whose lives may be bartered for the collective good. Rather, they should be regarded and treated compassionately, as subjects and unique individuals who have interests of their own (Table 2).

**Table 2:** Formal arguments in support of compassionate conservation that arise from the position that all sentient beings are persons

Critique of compassionate conservation	Response	Formal argument for compassionate conservation
The primary purpose of conservation is biodiversity protection.	Agreed, but biodiversity includes all life.	P1. The goal of conservation is to protect biodiversity. P2. Biodiversity includes all life. C. Therefore, the goal of conservation is to protect all life.
Conservation is already compassionate to animals.	Not according to the definition of *compassion* used with regard to persons.	P1. Conservation should exemplify compassion. P2. Exemplifying compassion entails following the Golden Rule in the treatment of all persons. C. Therefore, conservation should follow the Golden Rule in its treatment of all persons.
Conservation should prioritize compassion to humans.	Compassion should extend to all sentient beings.	P1. Conservation should treat persons with compassion. P2. All sentient animals are persons. C. Therefore, conservation should treat all sentient animals with compassion.

Although our main interest here is in underlying conceptual claims, we also question the empirical claim, expressed in P4, that harmful tactics are necessary to achieve existing conservation goals. Most lethal programs are not evidence based or even monitored (e.g., Reddiex & Forsyth 2007; Dubois et al. 2017; Doherty et al. 2019). Many lethal programs are known to fail or exacerbate extinction risk by disrupting social and trophic interactions (e.g., Wanless et al. 2007; Bergstrom et al. 2009; Wallach et al. 2010); curtailing emergent ecological dependencies (Schlaepfer et al. 2011); harming species that now thrive only outside their native ranges (Wallach et al. 2020); and overlooking the underlying human‐caused ecological changes shaping species interactions that result in extinctions (Doherty et al. 2015). Additionally, and importantly, the normalization of lethal programs crowds out motivation to invest in research on compassionate alternatives (Dubois et al. 2017).

## Response to Claim that Conservation is already Compassionate to Animals

Compassionate conservationists argue that only those conservation actions treating sentient beings with compassion are ethically appropriate (Wallach et al. 2018). Critics respond that invasive and lethal conservation programs are already compassionate because they minimize nonhuman suffering overall (Table 1). For example, Driscoll and Watson (2019) “want to preserve a morality that values endemic species and that goes beyond the bullet or bait to the unseen suffering caused by taking no action to control invasive species.” The first formalized argument is as follows:

P1. A conservation action is compassionate if it minimizes suffering. (ethical)
P2. Invasive and lethal conservation programs often minimize suffering. (empirical)
C. Therefore, invasive and lethal conservation programs are often compassionate.

It is important to carefully consider how compassion is being characterized. Critics who claim lethal conservation programs, such as poisoning foxes and aerial gunning wild horses (Driscoll & Watson 2019), are compassionate (Table 1) mean they minimize suffering of nonhuman animals that are harmed to achieve conservation objectives (e.g., by selecting faster acting poisons and sharper shooters and aiming to avert suffering that can arise in the absence of lethal programs) (Hampton et al. 2019). This is not how the term is applied in compassionate conservation.

Compassion literally means to suffer with. Emotionally, compassion engenders care and concern for the well‐being of others (Goetz et al. 2010). Ethically, compassion can be understood as a virtue: a disposition of good character manifested by receptivity and responsiveness (Sandler & Cafaro 2005). Compassion spurs one to recognize another as a person: as an intrinsically and uniquely valuable individual whose interests kindle one's concern and respect. It is helpful to think of a compassionate person as one who strives to follow the Golden Rule, a maxim of reciprocity (“treat others as you wish to be treated”) found in various forms across cultures, languages, religions, and ethical traditions (Küng 1993; Gensler 2013). A compassionate conservationist would generally strive to minimize suffering, but not by intentionally harming other persons. Indeed, no one would consider lethal control of human populations compassionate irrespective of the rationale, method, or even outcome. Critics, therefore, equivocate on the word *compassion* by offering a different and abridged meaning (Table 2).

Other critics assert that lethal conservation programs are appropriate where they decrease aggregate suffering, making their case on grounds of animal welfare consequentialism, rather than compassion (Table 1). It is beyond our scope here to make a case for compassion over (or perhaps alongside) consequentialism, but we dispute the claim that lethal conservation programs decrease overall suffering.

Conservation does not aim to reduce suffering per se, nor does it aim to change fundamental evolutionary processes, a view considered widely appropriate across animal and environmental ethics (e.g., Callicott 1988; Donaldson & Kymlicka 2011). Harm, pain, and death are integral to life. However, critics claim suffering is “[outside] natural evolutionary processes” (Driscoll & Watson 2019) when instigated by organisms whose occurrence or densities result from human activity. These critics also suggest that such forms of suffering can be minimized by lethal programs (Table 1). The second formalized argument is as follows:

P1. Populations augmented by human activity are unnatural. (ethical)
P2. Some unnatural populations increase suffering. (empirical)
C. Therefore, removing these unnatural populations will decrease suffering.

Premise 1 refers to wildlife populations that, due to human influence, are “deemed foreign, nonnative, invasive, or feral… and therefore harmful to biodiversity” (Hampton et al. 2019). Put differently, populations influenced by humans are considered external to biodiversity (Wallach et al. 2020). This notion presupposes a belief not only that humans are distinct from and outside of nature, but also that they have the power to transform otherwise natural (nonhuman) entities into a distinct class of unnatural (humanized) entities. In this way, all species are defined and valued based on their relationships with humans. Compassionate conservation rejects this premise, calling conservationists to decenter humans from the stories of nonhuman persons by recognizing that they have their own interests and experiences, independent of their interactions with humans (Table 2).

Premise 2 then claims that some unnatural populations cause increased suffering, of the sort that is of moral concern for humans (e.g., differentiating between a bird's suffering when predated upon by a native versus a non‐native cat). Premise 2 is difficult if not impossible to verify. Even if we accept the distinction between natural and unnatural populations, substantiating P2 would require comparing the amounts and types of suffering experienced by a range of organisms in similar systems, with and without the so‐called unnatural population. Although unique kinds of animal welfare harms can certainly occur in anthropogenically influenced ecological systems (e.g., Finn & Stephens 2017; Jiguet et al. 2019), there is no reason to assume a direct association between suffering and the unnaturalness of the system. Thus, rigorous evidence is lacking to support P2.

Finally, even if P1 and P2 were incontestable, they do not necessarily support the conclusion. As a basic rule of deduction, even when one knows if [A], then [B], one cannot infer if not [A], then not [B]. For example, just from the statement if [it rains], then [wet grass] one cannot conclude if [no rain], then [no wet grass] (maybe the sprinkler is running). Thus, from if [unnatural populations] then [more suffering], one cannot necessarily conclude, if [not unnatural populations] then [not more suffering]. The opposite is just as likely. Conservation control and eradication programs fracture social and trophic relationships, often perpetuating the harms these programs aim to resolve and creating additional and severe pain, trauma, and grief (e.g., Bradshaw et al. 2005; Ferdowsian et al. 2011; O'Neill et al. 2017).

## Response to Claim that Conservation Should Prioritize Compassion to Humans

Compassionate conservationists call for inclusion of all sentient beings in conservation's moral community (Ramp & Bekoff 2015; Wallach et al. 2018). Critics allege that such an approach would encourage indifference toward humans (Oommen et al. 2019). Their reasoning is summarized as follows:

P1. Conservation actions should treat humans with compassion. (ethical)
P2. Treating nonhumans with compassion can preclude treating humans with compassion. (empirical)
C. Therefore, in these cases, conservation actions should not treat nonhumans with compassion.

Premise 1 is uncontroversial among compassionate conservationists and their critics. We agree that conservationists should demonstrate compassion for humans by refraining from actions that infringe on their vital interests. However, Oommen et al. (2019) acknowledge no parallel requirement to treat sentient nonhuman beings with compassion, which in their view would express “moral extensionism or humanization of wild animals and the artificial attribution of moral standing to nonhuman[s]” (Oommen et al. 2019). This critique arbitrarily restricts moral standing to members of *Homo sapiens*, discounting unequivocal scientific evidence that many nonhuman animals possess morally relevant traits, including not only sentience, but also, for example, intelligence, emotion, self‐awareness, and the ability to form meaningful relationships (Bekoff & Pierce 2017). Recognition of these qualities and their ethical implications is increasingly influencing society (e.g., van Eeden et al. 2019; Manfredo et al. 2020). Thus, we would respond to Oommen et al. (2019) that failure to engage in “moral extensionism” signifies a dogmatic denial of evidence.

Premise 2 alleges that compassion for other sentient beings can foster misanthropy or apathy toward human suffering. To back this claim, critics point to cases where animal protection is used to advance oppressive and violent political regimes (Oommen et al. 2019). These examples are red herrings. The mobilization of so‐called animal protection for nationalistic and racist purposes is premised on the objectification of animals, not compassion for animals (Narayanan 2019).

Evidence suggests that humans who disparage or violate animals are more likely to treat humans similarly. Both philosophical (Horta 2010) and psychological research has associated human exceptionalism with other prejudices (e.g., racism). Human exceptionalism and interhuman prejudices can be mutually reinforcing, possibly because both are predicated on upholding strict hierarchies and binary social categorizations (Jackson 2019). Caviola et al. (2019) found positive correlations between human exceptionalism and racism, sexism, and homophobia and negative correlations with empathetic concern. Dhont et al. (2014) found that a social dominance orientation drives supremacist attitudes toward both nonhuman animals and ethnic minorities. These studies caution that objectifying nonhuman animals can counteract both human and nonhuman rights agendas (Kymlicka 2018). Park and Valentino (2019) found that support for animal rights is associated with higher levels of support for the human rights of disadvantaged groups at individual and state policy levels, suggesting that moral concern for humans and other animals is mutually reinforcing.

A world with few moral persons is easier to navigate because any conflict between competing interests can be brought to a swift resolution by prioritizing those few who are included in one's moral community. A more inclusive moral community creates a more complex moral terrain. Compassionate conservation recognizes a moral community populated by human and nonhuman persons, aiming to attend to all persons and their claims, even when they conflict (Table 2). Certainly, in some situations, it can be challenging to find ideal solutions. Even in the strictly human domain, it can be difficult or impossible to fulfill all moral obligations (Batavia et al. 2020). However, the conclusion that conservationists should simply renounce compassion for nonhuman animals in cases of conflict is inconsistent with the understanding of compassion as a virtue. It makes no sense to suggest a person who is compassionate by disposition should selectively withhold compassion in cases of conflict. Compassion becomes even more important under such circumstances as a caring response to any harm regretfully enacted against fellow persons.

## Discussion

Although the belief that nonhuman animals have some moral standing may be broadly shared among conservationists, compassionate conservation is distinguished by the recognition of nonhuman personhood. Proponents call to include all sentient beings as persons in conservation's moral community through the cultivation of compassion (Ramp & Bekoff 2015; Wallach et al. 2018). Critics of compassionate conservation generally deny the personhood of all beings but humans by calling for the continuation of programs that harm sentient beings, who are often intelligent, emotional, and social, for the perceived greater good of conservation.

On scientific and ethical grounds, there are good reasons to extend personhood to nonhuman animals (Midgley 1985; Rose 2011; Dayan 2018). The burden of proof should no longer lie with those who seek to expand conservation's moral community, but with those who wish to enforce narrow boundaries (Laham 2009). For compassionate conservationists, sentience is sufficient grounds to recognize personhood. Others may believe that different qualities are morally relevant, and we invite ongoing dialogue on this important topic. But as a starting point, personhood should not be a status automatically limited to humans. Holding humans separate and aloft from the rest of the living world has legitimated the historic and ongoing exploitation of the more‐than‐human world, which is arguably the reason conservation was needed in the first place (Plumwood 1993).

Opposition to compassionate conservation is often linked to the legitimate concern that at times conservationists are faced with difficult choices: harm individuals or lose species (Rohwer & Marris 2019). Under such tragic circumstances, it is not clear that any decision can be made with moral impunity (Batavia et al. 2020). Our most quotidian moments harm sentient beings, and choices must be made that inevitably prioritize some over others. How then is one to act ethically if every act holds the potential to harm fellow persons? There is no easy answer (Batavia et al. 2020). But if one takes seriously the notion that all sentient beings are persons, forming and pursuing conservation objectives founded on mass killing would become inconceivable. The default of domination would be replaced with a default of compassion. This does not mean that one never harms a person nor that there cannot be variations in our obligations to different persons (Plumwood 2008; Robinson 2014). Between perfectly equal moral status for all and categorical moral segregation of the few lies a wide expanse where a more inclusive and contextual moral terrain can be explored.

Conservationists who restrict personhood to humans may still attribute other animals some degree of moral standing. For example, Hayward et al. (2019) state, “most mainstream conservationists are keen to embrace ethical concern for individual animals as an important element in conservation best practices, but only to the extent that it is consistent with landscape‐level methods of protecting native biodiversity.” In other words, the “compassionate tail [should not] wag the conservation dog” (Hayward et al. 2019). For compassionate conservationists, this is not good enough. Relegating compassion to a virtue to be dragged behind action (or worse, to be docked) does little to limit the entrenched violence regularly enacted against sentient beings in conservation programs. Beyond simply replacing lethal tools with nonlethal tools to achieve the same ends, compassionate conservation challenges the very agendas and logics underlying conservation. For example, rather than merely asking how biodiversity can be protected from feral cats with nonlethal tools, one is able to ask what is revealed when feral cats are accepted as part of biodiversity (Wallach et al. 2020).

That no clean biological or evolutionary boundary separates humans from other animals is widely accepted, yet a stark ethical dualism persists, and abandoning it remains an almost unthinkable proposition. Some suggest that compassionate conservation is too subversive to even be allowed space at the table, going so far as to proclaim that “compassionate conservation is not conservation” (Driscoll & Watson 2019). Such a diktat risks harming the open exchange of ideas on which scholarship depends. If conservation's sole purpose is to protect native ecological collectives with little regard for other moral claims, then it is fair to say that neither compassionate conservation, the wider academic community, nor prevailing social values are aligned with conservation (van Eeden et al. 2019; Gbedomon et al. 2020; Manfredo et al. 2020). The time has come to change this entrenched definition of conservation. As Deborah Bird Rose (2011) said, “animals haunt the Western imagination, a haunting entailed by and sustained through our long‐lasting, but now crumbling, dualisms.”

Prevailing social values are shifting to align with views promoted by compassionate conservation (Manfredo et al. 2020). The moral recognition of personhood for nonhuman animals is even beginning to influence law. In 2019, an orangutan named Sandra was the first to be released from a zoo following Argentina's ground‐breaking legal recognition of nonhuman personhood. Addressing the press, Judge Elena Liberatori stated, “with that ruling I wanted to tell society something new: that animals are sentient beings and that the first right they have is our obligation to respect them” (BBC 2019). The implications of these societal shifts are not trivial for conservation.

Compassionate conservation is not a challenge to conservation per se, but a good‐faith response to growing societal recognition worldwide that nonhuman animals feel, that they have lives, experiences, and relationships that matter to them, and that should matter to us (e.g., European‐Parliament 2010; Kansal 2016; Africa‐Union 2017; Bruskotter et al. 2019; van Eeden et al. 2019; Manfredo et al. 2020). It is not farfetched to suggest that changing social values makes the transition to more compassionate forms of conservation unavoidable. Compassion sits at the heart of many religious and ethical traditions—not because it is obvious or simple, but precisely because it is difficult and demanding. We embrace compassion for its ability to bridge between ourselves and Earth's great diversity of persons. Compassionate conservation offers a way forward, to seize the challenges and opportunities that rise in the dust of our crumbling dualisms.

## References

[cobi13494-bib-0001] Africa‐Union . 2017 The animal welfare strategy in Africa. Africa‐Union, Nairobi, Kenya.

[cobi13494-bib-0002] Batavia C , Nelson MP . 2017 For goodness sake! What is intrinsic value and why should we care? Biological Conservation 209:366–376.

[cobi13494-bib-0003] Batavia C , Nelson MP , Wallach AD . 2020 The moral residue of conservation. Conservation Biology. 10.1111/cobi.13463. 31953967

[cobi13494-bib-0004] BBC . 2019 Orangutan with human rights to begin new life in Florida. Available from https://www.bbc.com/news/world-us-canada-49856859 (accessed January 2020).

[cobi13494-bib-0005] Bekoff M , Pierce J . 2017 The animals' agenda: freedom, compassion, and coexistence in the human age. Beacon Press, Boston, Massachusetts.

[cobi13494-bib-0006] Bergstrom DM , Lucieer A , Kiefer K , Wasley J , Belbin L , Pedersen TK , Chown SL . 2009 Indirect effects of invasive species removal devastate World Heritage Island. Journal of Applied Ecology 46:73–81.

[cobi13494-bib-0007] Bhattacharyya J , Larson BM . 2014 The need for indigenous voices in discourse about introduced species: insights from a controversy over wild horses. Environmental Values 23:663–684.

[cobi13494-bib-0008] Bradshaw GA , Schore AN , Brown JL , Poole JH , Moss CJ . 2005 Elephant breakdown. Nature 433:807–807. 15729320 10.1038/433807a

[cobi13494-bib-0009] Bruskotter JT , Vucetich JA , Dietsch A , Slagle KM , Brooks JS , Nelson MP . 2019 Conservationists’ moral obligations toward wildlife: values and identity promote conservation conflict. Biological Conservation 240:108296.

[cobi13494-bib-0010] Callicott JB . 1988 Animal liberation and environmental ethics: back together again. Between the Species 4:3.

[cobi13494-bib-0011] Caviola L , Everett JA , Faber NS . 2019 The moral standing of animals: towards a psychology of speciesism. Journal of Personality and Social Psychology 116:1011–1029. 29517258 10.1037/pspp0000182

[cobi13494-bib-0012] Chew MK , Hamilton AL . 2011 The rise and fall of biotic nativeness: a historical perspective Pages 35–48 in Richardson DM, editor. Fifty years of invasion ecology: the legacy of Charles Elton. Wiley‐Blackwell, United Kingdom.

[cobi13494-bib-0013] Darwin C . 1871 The descent of man and selection in relation to sex. John Murray, London.

[cobi13494-bib-0014] Dayan C . 2018 Personhood Pages 267–279 in Gruen L, editor. Critical terms for animal studies. University of Chicago Press, Chicago, Illinois.

[cobi13494-bib-0015] Dhont K , Hodson G , Costello K , MacInnis CC . 2014 Social dominance orientation connects prejudicial human–human and human–animal relations. Personality and Individual Differences 61:105–108.

[cobi13494-bib-0016] Doherty T , Driscoll DA , Nimmo DG , Ritchie EG , Spencer R‐J . 2019 Conservation or politics? Australia's target to kill 2 million cats. Conservation Letters 12:e12633.

[cobi13494-bib-0017] Doherty TS , Dickman CR , Nimmo DG , Ritchie EG . 2015 Multiple threats, or multiplying the threats? Interactions between invasive predators and other ecological disturbances. Biological Conservation 190:60–68.

[cobi13494-bib-0018] Donaldson S , Kymlicka W . 2011 Zoopolis: a political theory of animal rights. Oxford University Press, United Kingdom.

[cobi13494-bib-0019] Driscoll DA , Watson MJ . 2019 Science denialism and compassionate conservation: response to Wallach et al. 2018. Conservation Biology 33:777–780. 30629767 10.1111/cobi.13273

[cobi13494-bib-0020] Dubois S , et al. 2017 International consensus principles for ethical wildlife control. Conservation Biology 31:753–760. 28092422 10.1111/cobi.12896

[cobi13494-bib-0021] European‐Parliament . 2010 Directive 2016/63/EU of the European Parliament and of the Council of 22 September 2010 on the Protection of Animals Used for Scientific Purposes. Official Journal of the European Union.

[cobi13494-bib-0022] Ferdowsian HR , Durham DL , Kimwele C , Kranendonk G , Otali E , Akugizibwe T , Mulcahy J , Ajarova L , Johnson CM . 2011 Signs of mood and anxiety disorders in chimpanzees. PLOS ONE 6 (e19855) 10.1371/journal.pone.0019855. 21698223 PMC3116818

[cobi13494-bib-0023] Finn H , Stephens N . 2017 The invisible harm: land clearing is an issue of animal welfare. Wildlife Research 44:377–391.

[cobi13494-bib-0024] Gbedomon RC , Salako VK , Schlaepfer MA . 2020 Diverse views among scientists on non‐native NeoBiota 54:49–69.

[cobi13494-bib-0025] Gensler HJ . 2013 Ethics and The Golden Rule. Routledge, New York.

[cobi13494-bib-0026] Goetz JL , Keltner D , Simon‐Thomas E . 2010 Compassion: an evolutionary analysis and empirical review. Psychological Bulletin 136:351. 20438142 10.1037/a0018807 PMC2864937

[cobi13494-bib-0027] Goodall J . 1998 Essays on science and society: learning from the chimpanzees: a message humans can understand. Science 282:2184–2185.

[cobi13494-bib-0028] Hampton JO , Warburton B , Sandøe P . 2019 Compassionate versus consequentialist conservation. Conservation Biology 33:751–759. 10.1111/cobi.13249 30411399

[cobi13494-bib-0029] Haraway DJ . 2013 When species meet. University of Minnesota Press, Minneapolis, Minnesota.

[cobi13494-bib-0030] Hayward MW , et al. 2019 Deconstructing compassionate conservation. Conservation Biology 33:760–768. 31206825 10.1111/cobi.13366

[cobi13494-bib-0031] Hill E . 2013 Archaeology and animal persons: toward a prehistory of human–animal relations. Environment and Society 4:117–136.

[cobi13494-bib-0069] Horta O . 2010 What is speciesism? Journal of Agricultural and Environmental ethics 23:243–266.

[cobi13494-bib-0032] Hughes W , Lavery J , Doran K . 2010 Critical thinking: an introduction to the basic skills. 6th edition Broadview Press, Peterborough.

[cobi13494-bib-0033] Jackson LM . 2019 Speciesism predicts prejudice against low‐status and hierarchy‐attenuating human groups. Anthrozoös 32:445–458.

[cobi13494-bib-0034] Jiguet F , Sunnen L , Prévot A‐C , Princé K . 2019 Urban pigeons loosing toes due to human activities. Biological Conservation 240:108241.

[cobi13494-bib-0035] Kansal V . 2016 The curious case of Nagaraja in India: are animals still regarded as “property” with no claim rights? Journal of International Wildlife Law & Policy 19:256–267.

[cobi13494-bib-0036] Küng H, editor. 1993 A global ethic. The declaration of the Parliament of the World's Religions. Available from https://www.global-ethic.org/declaration-toward-a-global-ethic/ (accessed January 2020).

[cobi13494-bib-0037] Kymlicka W . 2018 Human rights without human supremacism. Canadian Journal of Philosophy 48:763–792.

[cobi13494-bib-0038] Laham SM . 2009 Expanding the moral circle: inclusion and exclusion mindsets and the circle of moral regard. Journal of Experimental Social Psychology 45:250–253.

[cobi13494-bib-0039] Low P , Panksepp J , Reiss D , Edelman D , Van Swinderen B , Koch C . 2012 The Cambridge declaration on consciousness. Francis Crick Memorial Conference, Cambridge, United Kingdom.

[cobi13494-bib-0040] Manfredo MJ , Urquiza‐Haas EG , Carlos AWD , Bruskotter JT , Dietsch AM . 2020 How anthropomorphism is changing the social context of modern wildlife conservation. Biological Conservation 241 10.1016/j.biocon.2019.108297.

[cobi13494-bib-0041] Marris E . 2013 Rambunctious garden: saving nature in a post‐wild world. Bloomsbury Publishing, New York.

[cobi13494-bib-0042] Midgley M . 1985 Persons and non‐persons Pages 52–62 in Singer P, editor. In defense of animals. Blackwell, New York.

[cobi13494-bib-0043] Narayanan Y . 2019 “Cow is a mother, mothers can do anything for their children!” Gaushalas as landscapes of anthropatriarchy and Hindu patriarchy. Hypatia 34:195–221.

[cobi13494-bib-0044] Nelson MP , Bruskotter JT , Vucetich JA , Chapron G . 2016 Emotions and the ethics of consequence in conservation decisions: lessons from Cecil the Lion. Conservation Letters 9:302–306.

[cobi13494-bib-0045] Nussbaum MC . 1995 Objectification. Philosophy & Public Affairs 24:249–291.

[cobi13494-bib-0046] O'Neill AJ , Cairns KM , Kaplan G , Healy E . 2017 Managing dingoes on Fraser Island: culling, conflict, and an alternative. Pacific Conservation Biology 23:4–14.

[cobi13494-bib-0047] Oommen MA , et al. 2019 The fatal flaws of compassionate conservation. Conservation Biology 33:784–787. 30977162 10.1111/cobi.13329

[cobi13494-bib-0048] Park YS , Valentino B . 2019 Animals are people too: explaining variation in respect for animal rights. Human Rights Quarterly 41:39–65.

[cobi13494-bib-0049] Plumwood V . 1993 Feminism and the mastery of nature. Routledge, New York.

[cobi13494-bib-0050] Plumwood V . 2008 Tasteless: towards a food‐based approach to death. Environmental Values 17:323–330.

[cobi13494-bib-0051] Ramp D , Bekoff M . 2015 Compassion as a practical and evolved ethic for conservation. BioScience 65:323–327.

[cobi13494-bib-0052] Reddiex B , Forsyth DM . 2007 Control of pest mammals for biodiversity protection in Australia. II. Reliability of knowledge. Wildlife Research 33:711–717.

[cobi13494-bib-0053] Robinson M . 2014 Animal personhood in Mi'kmaq perspective. Societies 4:672–688.

[cobi13494-bib-0054] Rohwer Y , Marris E . 2019 Clarifying compassionate conservation with hypotheticals: response to Wallach et al. 2018. Conservation Biology 33:781–783. 30653251 10.1111/cobi.13274

[cobi13494-bib-0055] Rose DB . 2011 Wild dog dreaming: love and extinction. University of Virginia Press, Charlottesville, Virginia.

[cobi13494-bib-0056] Russell JC , et al. 2016 Importance of lethal control of invasive predators for island conservation. Conservation Biology 30:670–672. 26634637 10.1111/cobi.12666

[cobi13494-bib-0057] Sandbrook C , Fisher JA , Holmes G , Luque‐Lora R , Keane A . 2019 The global conservation movement is diverse but not divided. Nature Sustainability 2:316–323.

[cobi13494-bib-0058] Sandler R , Cafaro P . 2005 Environmental virtue ethics. Rowman & Littlefield Publishers, Lanham, Maryland.

[cobi13494-bib-0059] Schlaepfer MA , Sax DF , Olden JD . 2011 The potential conservation value of non‐native species. Conservation Biology 25:428–437. 21342267 10.1111/j.1523-1739.2010.01646.x

[cobi13494-bib-0060] Srinivasan K , Kasturirangan R . 2017 Conservation and invasive alien species: violent love Pages 433–452 in Maher J, Pierpoint H, Beirne P, editors. The Palgrave international handbook of animal abuse studies. Palgrave Macmillan, London, United Kingdom.

[cobi13494-bib-0061] van Eeden LM , Newsome TM , Crowther MS , Dickman CR , Bruskotter J . 2019 Social identity shapes support for management of wildlife and pests. Biological Conservation 231:167–173.

[cobi13494-bib-0062] Vucetich JA , Nelson MP . 2007 What are 60 warblers worth? Killing in the name of conservation. Oikos 116:1267–1278.

[cobi13494-bib-0063] Wallach AD , Bekoff M , Batavia C , Nelson MP , Ramp D . 2018 Summoning compassion to address the challenges of conservation. Conservation Biology 32:1255–1265. 29700860 10.1111/cobi.13126

[cobi13494-bib-0064] Wallach AD , Bekoff M , Nelson MP , Ramp D . 2015 Promoting predators and compassionate conservation. Conservation Biology 29:1481–1484. 25976274 10.1111/cobi.12525

[cobi13494-bib-0065] Wallach AD , Johnson CN , Ritchie EG , O'Neill AJ . 2010 Predator control promotes invasive dominated ecological states. Ecology Letters 13:1008–1018. 20545732 10.1111/j.1461-0248.2010.01492.x

[cobi13494-bib-0066] Wallach AD , et al. 2020 When all life counts in conservation. Conservation Biology 10.1111/cobi.13447. 31782203

[cobi13494-bib-0067] Wanless RM , Angel A , Cuthbert RJ , Hilton GM , Ryan PG . 2007 Can predation by invasive mice drive seabird extinctions? Biology Letters 3:241–244. 17412667 10.1098/rsbl.2007.0120 PMC2464706

